# Development and Preliminary Evaluation of an EfficientNet-Based Deep Learning System for Ultrasound Assessment of Neck Disorders: A Single-Center Study

**DOI:** 10.3390/diagnostics16050728

**Published:** 2026-03-01

**Authors:** Wei Ding Wang, Siew-Ying Mok, Yang Mooi Lim, Hui Saan Tham, Lee Fan Tan, Chai Nien Foo, Clara Pei Ying Lim, Choon-Hian Goh

**Affiliations:** 1 Department of Mechatronics and Biomedical Engineering, Lee Kong Chian Faculty of Engineering and Science, Universiti Tunku Abdul Rahman, Bandar Sungai Long, Kajang 43000, Selangor, Malaysiamoksy@utar.edu.my (S.-Y.M.); tanlf@utar.edu.my (L.F.T.); 2 Center for Cancer Research, M. Kandiah Faculty of Medicine and Health Sciences, Universiti Tunku Abdul Rahman, Bandar Sungai Long, Kajang 43000, Selangor, Malaysia; ymlim@utar.edu.my (Y.M.L.); foocn@utar.edu.my (C.N.F.); 3 HAN Neuro Acupuncture & Herbal Specialists, Cyberjaya 63000, Selangor, Malaysia; thamtcm@gmail.com (H.S.T.); clpying92@gmail.com (C.P.Y.L.)

**Keywords:** EfficientNet, transfer learning, texture analysis, classification, ultrasound, neck disorder

## Abstract

**Background/Objectives**: Neck disorders encompass a range of discomforts impacting a person’s quality of life. Traditional diagnostic methods, such as physical tests and imaging techniques, rely heavily on clinician expertise, leading to potential variability in assessments. While ultrasound imaging is commonly used, the application of machine learning models to assess neck disorders, particularly fascial abnormalities, remains limited. This study seeks to fill this gap by developing a machine learning model using ultrasound images to provide accurate and efficient support for diagnosing neck disorders. **Methods**: Due to limited availability of labeled ultrasound data for neck disorders, developing robust and generalizable models remains a challenge. In this study, a neck disorder assessment system was developed using ultrasound images collected from 184 patients by employing various machine learning algorithms. To address data scarcity and improve model generalizability, an approach utilizing EfficientNet with transfer learning was introduced and thoroughly assessed using the trained model on a completely clean test dataset, ensuring the robustness of the solution. The model was trained using 5-fold cross-validation with the respective weight of each class and AdamW as the optimizer. **Results**: The results showed promising performance, with the deep fascia fuzzy texture and deep fascia and myofascial adhesion at lower cervical regions demonstrating the highest weighted average F1-scores of 76% and 81%, respectively. The macro averages reflected similar performance, at 74% and 78%, respectively, indicating consistent class-wise accuracy for these regions. **Conclusions**: The proposed model demonstrated robust classification performance for neck disorder assessment, particularly in evaluating the lower cervical region. This approach has the potential to support clinical decision-making by providing consistent, efficient, and accurate diagnostic assistance. Further refinement and validation across diverse clinical settings will be critical to enhance its real-world applicability.

## 1. Introduction

Neck disorders can encompass a wide range of conditions, including muscular and musculoskeletal neck pain. These disorders could lead to discomfort, pain, and restricted neck motion. Neck disorders have several definitions based on the anatomical location and other considerations [1]. As technology evolves, people are increasingly dependent on electronic devices, adopting improper postures that can result in varying degrees of neck damage [2,3,4]. By 2050, the global number of neck pain cases is expected to reach 269 million, reflecting a 32.5% increase compared to 2020 [5]. This growing prevalence underscores the critical need for innovative solutions to support the timely and accurate diagnosis of neck disorders.

As a result, multiple diagnostic techniques have been introduced to diagnose the disorders with high accuracy, including physical tests, ultrasound imaging, computed tomography (CT), and magnetic resonance imaging (MRI). Additionally, the cervical range of motion (CROM) device can be used for physical tests to assess the muscle capability of the patients with reference to their ages and body indices [6]. Since physical test results are subject to the pain tolerance of a patient, radiology has revolutionized the way for diagnosis. Ultrasound imaging is getting popular due to its non-invasive nature and ability to provide real-time visualization of soft tissues and structures in the neck region, making it useful in detecting the fascial characteristics of a patient for diagnosis [7]. Fascia screening provides useful information for neck disorders, such as deep fascia discontinuity and adhesion [8]. However, a clinician requires extensive training to analyze the images and categorize the severity of the neck disorder. While some studies have applied machine learning and deep learning approaches to related musculoskeletal ultrasound tasks—such as fascia segmentation using deep networks [9] and texture-based classification of myofascial trigger points [10]—there is no prior work specifically focused on models for ultrasound-based neck fascia analysis. Hence, this study aimed to develop machine learning algorithms for assessing neck disorder severity from ultrasound images, which could assist clinicians in performing preliminary analyses before integrating them with other information to make informed decisions.

Casaletto et al. [11] demonstrated the efficacy of ultrasound in visualizing neck nerves for clinical diagnosis, providing a foundation for understanding ultrasound clinical utility in nerve imaging. In addition, techniques like transfer learning—which adapts pretrained models to smaller, specialized datasets [12]—have been explored in ultrasound research to enhance diagnostic accuracy in tumor detection. For example, Cheng and Malhi [13] evaluated transfer learning using CaffeNet and VGGNet for an abdominal ultrasound dataset, achieving the highest test accuracy of 77.9% compared to 71.7% accuracy by the radiologist. Similarly, Gu et al. [14] proposed a neural network-based classification method for medical ultrasound image processing, emphasizing the role of convolutional architectures and transfer learning to improve diagnostic accuracy in the detection of thyroid nodules. Saha and Sheikh [15] utilized data augmentation and the Auxiliary Classifier Generative Adversarial Network (ACGAN) for classifying breast ultrasound images, which consisted of 150 malignant images and 100 benign images. Despite the highly imbalanced dataset, the authors achieved 98.8% accuracy, compared to 96.4% with VGG19.

EfficientNet, a deep neural network, utilizes highly effective compound scaling, which optimizes the model’s width, depth, and resolution to maximize performance while minimizing computational cost [16]. EfficientNet V2, an enhanced version introduced by Tan and Le [17], improves the original model with faster training speeds and better parameter efficiency. Marques et al. [18] developed an automated medical diagnosis system for Coronavirus disease (COVID-19) with CT X-ray lung images. They achieved an average F1-score of 97.11% for multiclass classification and 99.62% for binary classification.

This study addresses the challenge of automating neck disorder assessment using ultrasound imaging by applying transfer learning techniques. A curated dataset of ultrasound images from 184 patients was developed, focusing on muscular neck conditions with varied texture patterns—an area with limited representation in existing datasets. Unlike tumor detection, these images present a unique challenge due to their varied textures and lack of tumor-specific features. This makes them distinctly different from the more homogeneous, tumor-focused datasets, presenting a more complex task for accurate classification. EfficientNet, a high-performing convolutional neural network (CNN) architecture, was adapted to this domain to classify ultrasound features associated with neck disorders. The study contributes to the field by demonstrating the feasibility of applying deep learning to ultrasound analysis of neck fascia and by providing a foundation for future research in clinical decision support systems for neck disorder diagnosis. In particular, this work represents the first application of an EfficientNet-based deep learning model for fascia-texture classification using ultrasound images, addressing an area of musculoskeletal assessment that has been largely unexplored in prior literature.

## 2. Materials and Methods

**Figure 1:**
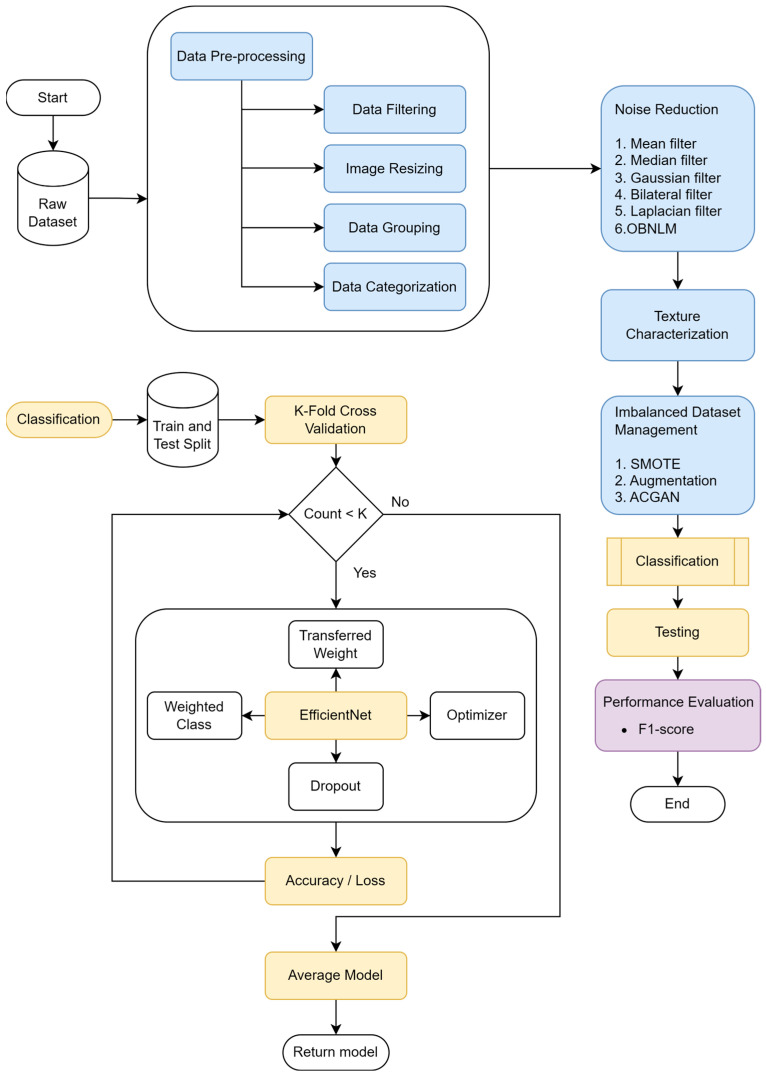
The Workflow Diagram of This Study. The figure illustrates the complete pipeline, including data preprocessing, noise reduction, texture characterization, and imbalanced dataset management. The figure explicitly differentiates between the two evaluation strategies employed in this study: (i) a train–test split, used for benchmarking with external ultrasound datasets, and (ii) K-fold cross-validation, applied exclusively to the neck ultrasound dataset to ensure robust model assessment given the limited sample size. The training stage incorporates transfer learning using EfficientNet with class-weighting, dropout, and optimization. Model performance is evaluated using F1-scores.

The proposed solution consists of several stages, including data preprocessing and classification. The classification models were developed using two different platforms. The first was a desktop equipped with an NVIDIA RTX 3080Ti GPU (NVIDIA Corporation, Santa Clara, CA, USA), which has 12 GB of CUDA memory. The second device was Google Colab Pro, utilizing an A100 GPU with 40 GB of CUDA memory. Figure 1 illustrates the workflow of the developed solution, detailing each stage from data preprocessing to final classification.

### 2.1. Dataset

The data were collected from HAN Neuro Acupuncture & Herbal Specialists Sdn. Bhd. with ethical approval from the UTAR Scientific and Ethical Review Committee (SERC), reference number U/SERC/271/2021. The data collection period spanned from November 2022 to October 2024. The study included subjects who met the following inclusion criteria: no skin diseases or injuries on the limbs, no skin allergies, and no incompatibility with ECG electrodes. All participants were fully informed of the examination procedures and provided written informed consent prior to their participation. Exclusion criteria included patients with any of the following conditions: dermatological lesions on the limbs, excessive perspiration (hyperhidrosis), the use of a cardiac pacemaker or any implanted electronic devices such as defibrillators, inability to sit during the examination, metal pins or prostheses on the extremities or joints, pregnancy, or the absence of one or more limbs. Importantly, patients with prior neck surgery or trauma were not included in the study to prevent confounding factors.

All images were acquired using a Mindray DC-70 (Shenzhen Mindray Bio-Medical Electronics Co., Ltd., Shenzhen, Guangdong, China) high-resolution ultrasound system with Sound Touch Quantification. A linear array transducer (L12-3E) operating within a 3–12 MHz frequency range was used, with the acquisition set to F H10.0 (10 MHz high-frequency) in B-mode. The exported images had a resolution of 1260 × 910 pixels at 96 dpi. All scans were obtained under consistent protocol by two trained clinicians to ensure uniformity of image quality. The clinician provided a neck ultrasound dataset followed by ultrasound images analysis on each image. The data were collected from a total of 184 patients, comprising 75 males and 109 females, with informed consent. The males had an average age of approximately 43 years, weighed 74 kg, were 170 cm tall, and had a body mass index (BMI) of 26.3. The females had an average age of approximately 40 years, weighed 59 kg, were 160 cm tall, and had a BMI of 23.6. A two-tailed independent *t*-test showed no significant difference in age between males and females, but weight, height, and BMI were significantly higher in males compared to females. The summary of the analysis is presented in Table 1.

**Figure 2:**
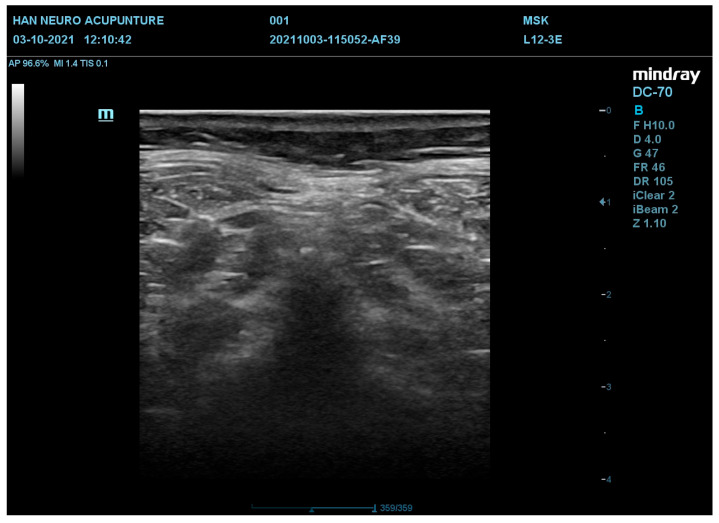
A representative raw ultrasound image illustrating key features: deep fascia discontinuity (score = 2), deep fascia “Wen Li Bu Qing” (score = 1), Modified Heckmatt Scale (MHS) feature (score = 3), and deep fascia with myofascial adhesion (score = 1).

The ultrasound images were taken from three body parts, the cervical lower region (CL), cervical middle region (CM), and cervical upper region (CU), corresponding to C5, C3, and C1 in the cervical vertebrae, respectively. The features include deep fascia discontinuity, deep fascia “*wen li bu qing*” (fuzzy texture of deep fascia), the modified Heckmatt scale (MHS), and deep fascia and myofascial adhesion. Each category was scored either zero or one, except for deep fascia discontinuity, which was scored from zero to two, and the modified Heckmatt scale, which ranged from one to four. Table 2 shows the number of images collected for each score in each category. Figure 2 shows an example of a CL ultrasound image, along with the annotated scores provided by the clinician for each feature.

### 2.2. Data Preprocessing Procedures

Data preprocessing is crucial to arrange the data properly before any further steps, preventing failure due to faulty data. In this study, the preprocessing was performed using Pandas, which was employed to read the CSV file, check for empty cells, and validate that all scores were within the correct bounds. The first step of data preprocessing involved filtering the data to remove patients with empty or abnormal scores. A total of two patients had empty or abnormal scores in the scoring provided by the clinician, so only a total of 182 images were used for model development. Next, the MHS scores were renumbered from 1–4 to 0–3 for easier processing. After filtering, the images were cropped and resized to remove the fixed frame and retain only the ultrasound portion, thereby reducing computational costs by eliminating unnecessary pixels. The cropped image size was set to 620 × 620 pixels, based on uniform resizing to maintain consistency across all images. The ultrasound images were then synchronized with the numbering in the analysis file. At this stage, the images were ready for processing and were categorized according to their different features and scores.

Next, we further addressed noticeable noise in the images, which was not useful for algorithm development. Noise reduction aimed to mitigate the unavoidable speckles in ultrasound images. Conventional noise reduction methods, including mean filter, median filter, and Gaussian filter, were employed. Additionally, the Optimized Blockwise Non-Local Means (OBNLM) from the GitHub repository was tested. In this study, the Peak Signal-to-Noise Ratio (PSNR) metric was chosen as it is widely used in image processing for assessing the quality of reconstructed or denoised images. PSNR provides a straightforward, quantitative measure of the difference between the original and processed images, focusing on pixel-level error, making it ideal for evaluating the effectiveness of the filtering techniques applied.

Since the neck ultrasound images were analyzed based on their texture, texture characterization was carried out to highlight the crucial textures, making the analysis more significant and easier. The Gray-Level Co-Occurrence Matrix (GLCM) has proven to be one of the best texture descriptors over the years [19]. The GLCM works by calculating how often pairs of pixels with specific bit values occur. Several statistics can be obtained from the matrix, such as contrast, correlation, energy, homogeneity, and mean. The GLCM was computed using a 5 × 5 kernel, a pixel distance of 1, and an angle of 0°. A 5 × 5 neighborhood was selected to preserve fine-scale fascia echotexture while reducing sensitivity to random speckles. A distance of 1 pixel is widely recommended in radiomics studies for capturing the most discriminative local spatial dependencies, while the 0° orientation aligns with the predominant horizontal organization of cervical fascia layers. This configuration enables stable and anatomically meaningful texture feature extraction, consistent with current radiomics practice [20,21,22].

Patients typically seek medical attention when they feel unwell; therefore the dataset was highly unbalanced with the majority class on the severe characteristics. Snider et al. [23] reported that standard augmentations and ensemble predictions boosted ultrasound classification accuracy. Several methods were employed to manage the imbalanced datasets. The images were augmented by random horizontal flips, random rotations within 10°, and random affine transformations. The augmented images were then concatenated with the original images to achieve a balanced dataset. This augmentation and concatenation process was carried out within each category, as the imbalance varied across categories. From the augmented images, a random subset was selected and concatenated to match the number of images in the major class.

In the classification algorithm, the weight for each class in a particular category was adjusted based on the ratio in the dataset. This adjustment influenced the learning weight of each class in the model, ensuring that the algorithm accounted for the class imbalance during training. In practice, this means that classes with fewer images were assigned higher weights, while those with more images received lower weights, allowing the model to pay proportionally more attention to under-represented categories. This approach helps prevent the classifier from being biased toward the majority class and supports more balanced learning across all classes. Formally, for a dataset with *C* classes and *n_i_* samples in class *i*, the class weight was computed as *w_i_* = *N*/(*C × n_i_*), where *N* denotes the total number of samples.

### 2.3. Classification

Given the complexity of ultrasound images, a deep learning model was essential for iteratively learning important features through backpropagation from the provided training set. The first step involved splitting the original dataset into training and testing sets with a ratio of 9:1. The training set was further split into training and validation sets, in an 8:2 ratio. Piffer et al. [24] systematically reviewed “small data” AI in medical imaging, finding that transfer learning and data augmentation are widely used to mitigate scarcity. To ensure reproducibility, the deep learning model was provided with inputs resized to 480 × 480 pixels. During training, a set of light augmentations was applied, including random horizontal flipping and random posterization (2-bit), followed by normalization using ImageNet mean and standard deviation. ImageNet normalization was retained to ensure compatibility with the pretrained network and to maintain stable feature representations during transfer learning. Validation and test images were only resized and normalized, without augmentation. The model was trained using a 5-fold cross-validation approach, with 20 epochs per fold (totaling 100 training epochs).

Each fold was trained using a batch size of 16, an initial learning rate of 0.0002, and a learning-rate scheduler that reduced the learning rate by a factor of 0.1 every five epochs. A dropout rate of 0.2 was incorporated to help reduce overfitting. The model was implemented in the PyTorch framework and optimized with the Adam optimizer using default momentum settings.

In addition to using the pretrained weights from EfficientNet, which was trained on the 1000-class ImageNet dataset, two other datasets were obtained from Kaggle to perform transfer learning. The first dataset was the ultrasound images of breast cancer by [25], consisting of two classes of 224 × 224 pixel images and approximately 8000 training images. The second dataset was the brain MRI tumor dataset by [26], comprising four classes of 512 × 512 pixel images.

The brain MRI dataset, with 2870 training images, was computationally expensive to train due to its large size. Therefore, the dataset was reduced to 100 images per class, totaling 400 images—a practical trade-off to enable feasible computation. Although reduced, the network was able to learn general low-level features, such as edges and textures, which were transferable to the neck ultrasound dataset. These pretrained weights served as initializations for the ultrasound task, accelerating convergence and improving final model performance compared to training from scratch, without causing underfitting. The different approaches tested are listed in Table 3.

However, a key constraint of this study is the lack of segmentation masks, which limited the model’s ability to focus on specific regions of interest, such as the fascia and muscle structures, in the ultrasound images. This limitation meant that the model had to rely on raw image data without precise localization, which could have affected the model’s ability to extract more targeted features.

### 2.4. Performance Evaluation

After developing the algorithm, its performance was evaluated using two different metrics: F1-score (also referred to as Dice coefficient) in terms of macro average and weighted average. F1-scores quantified the algorithm’s performance by calculating the harmonic mean of precision and recall, indicating the algorithm’s reliability. F1-scores were computed from a confusion matrix, which consisted of true positive (TP), true negative (TN), false positive (FP), and false negative (FN). Given the unbalanced nature of the dataset, the metrics were computed using both macro average (the unweighted mean of all per-class scores) and weighted average (which accounts for the class weight to address class imbalance) [27]. Macro average computed the metrics independently for each class, while weighted average considered each class’s contribution proportional to its size. Accuracy, a common metric, was not considered as it could be misleading due to the dominance of the majority class.

(1) $$\begin{array}{c} Macro\;Average\;F1-Score=\frac{1}{N}\;\;\sum_{i=0}^{N}\frac{{2TP}_{i}}{{2TP}_{i}+{FP}_{i}+{FN}_{i}}\; \end{array}$$

(2) $$\begin{array}{c} Weighted\;Average\;F1-Score=\sum_{i=0}^{N}\frac{w_{i}}{N}\times \frac{{2TP}_{i}}{{2TP}_{i}+{FP}_{i}+{FN}_{i}} \end{array}$$

where *N* is the total number of classes in the respective feature; *i* is the index of the class in the respective feature; $w_{i}$ is the number of samples in class *i*.

## 3. Results

### 3.1. Data Preprocessing Outcomes

**Figure 3:**
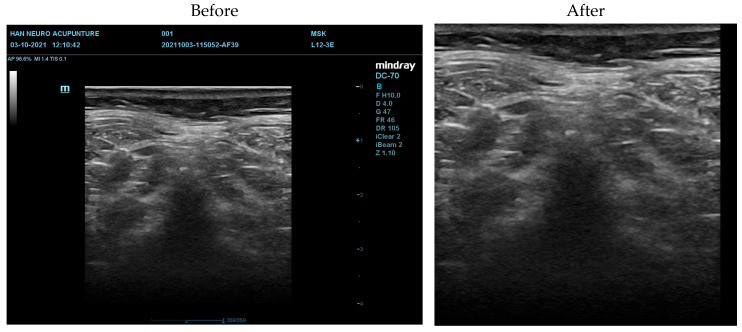
Images of a representative sample before and after cropping. The image was cropped from 1260 × 910 pixels to 620 × 620 pixels. This cropping step removes redundant background and ensures that the model focuses on the relevant anatomical region, improving consistency across samples.

The images were first cropped from 1260 × 910 pixels into 620 × 620 pixels, removing unnecessary portions that did not contain important features to enhance computational efficiency. A representative cropped image is shown in Figure 3.

**Figure 4:**
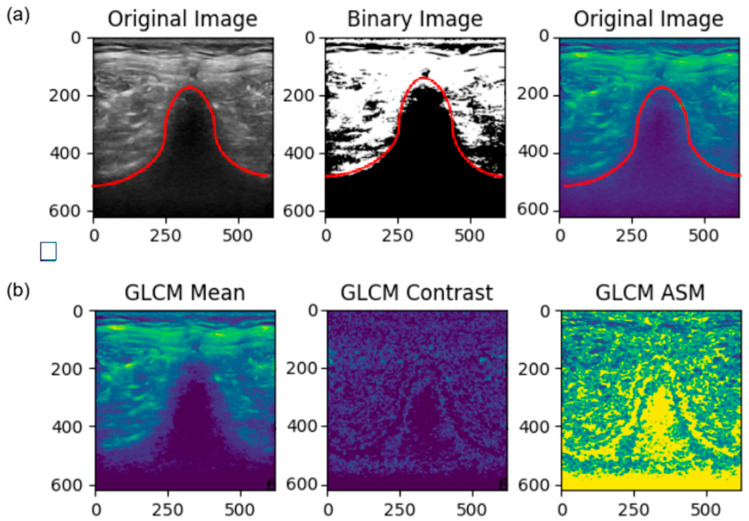
The Outputs of GLCM Texture Characterization. The ideal outcome of filtering was to remove the noise under the hill-shaped line (red color) while maintaining the muscle fiber texture. (**a**) The first image shows the raw image, the second image is the binary image, which highlights the areas of interest by separating the features from the background, and the third image represents the enhancement, with noise more visible. (**b**) Among GLCM mean, contrast, and ASM, the GLCM mean performed the best to remove noise while preserving the muscle fiber texture. The enhancement reduces entropy (6.43 → 4.01) and increases variance (1179.13 → 1647.90), indicating improved structural clarity and contrast separation. This preprocessing enhances texture clarity, supporting more reliable feature extraction by the model.

**Figure 5:**
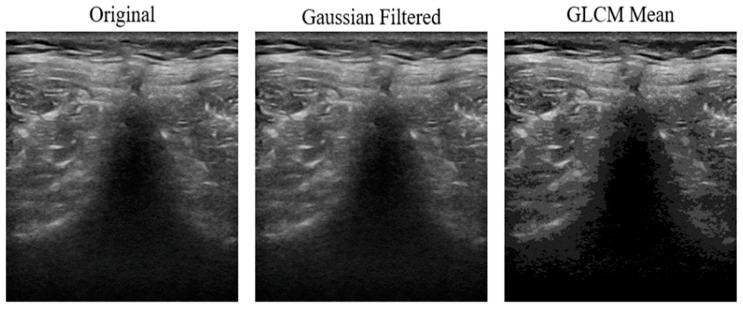
Comparison of The Image Before and After Preprocessing. The GLCM mean was done after the Gaussian Filter to better remove noise. The final output from the GLCM mean shows less noise and better contrast. This combined filtering pipeline improves fine structural details and reduces noise, resulting in clearer images that improve the reliability of downstream feature extraction.

Next, the images were filtered to reduce noise. The PSNR values for each filtering technique, ranked from highest to lowest, are as follows: Gaussian Filter (49.16 dB), Median Filter (43.14 dB), Bilateral Filter (42.04 dB), Mean Filter (39.45 dB), OBNLM (36.32 dB), and Laplacian Filter (21.76 dB) (see Appendix A.1). A higher PSNR indicates better image quality with less noise. Therefore, the Gaussian-filtered images were used for subsequent texture characterization. The GLCM-texturized images are shown in Figure 4. Among them, the GLCM mean images were chosen for further processing (see Appendix A.2 for more examples). The transition from the original image to the Gaussian-filtered image, and finally to the GLCM mean image, is shown in Figure 5. The GLCM mean image exhibited better contrast and eliminated noise in the pure black region, which helped to reduce image complexity.

### 3.2. Classification Algorithms

The different methods used for classification algorithms are shown in Table 4. Methods 1 to 6 were used to train on open datasets (the breast cancer dataset and the brain MRI dataset), with validation accuracy results presented. The breast cancer dataset was effectively trained using EfficientNet B0, achieving 99% validation accuracy, while the brain MRI dataset performed the best when trained with EfficientNet V2L, enhanced by the addition of a dropout layer with the probability of 0.2.

**Figure 6:**
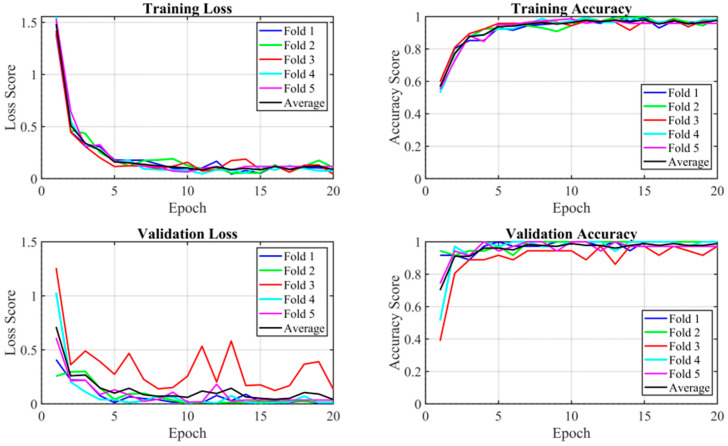
Learning Curves of The Best Model (Method 10).

Figure 6 shows an example of the learning curve for Method 10. The training loss curve converged rapidly until epoch 20 and then slowed and fluctuated at a very low loss, less than 0.2. The validation loss curve converged quickly in the first five epochs and then fluctuated between losses of 1.5 and 1.7. Similarly, in the accuracy curve graph, the training curve gradually increased and fluctuated between accuracies of 0.96 and 0.99, while the validation curve exhibited larger fluctuations. Early stopping was applied during training to prevent overfitting, ensuring that the model did not continue training once the validation loss stopped improving in 5 epochs.

The best-performing trained weights were used as the pretrained weights to train the cervical dataset. Various combinations were tested, and the results are shown in Table 5. During the model development process, to save computational cost and time, the models were first trained and tested on deep fascia fuzzy texture. If the model performed well, training proceeded with deep fascia discontinuity, followed by deep fascia and myofascial adhesion, and finally MHS. This stepwise procedure represents a structured incremental approach, starting from the easiest-to-learn feature and progressively moving to more challenging features. In the absence of a segmentation mask, deep fascia fuzzy texture was easier to learn by the neural network, as it contains texture-like features, compared to deep fascia discontinuity, which focuses on the discontinuity of the muscle fiber. Adhesion is also associated with muscle fiber texture, so we expected it to resemble deep fascia fuzzy texture. MHS, with the greatest number of classes, had the fewest images per class, making it the most challenging to process.

Table 5 presents the F1-scores for all tested classification algorithms. Methods 9 and 10 employed K-fold cross validation, with the training and validation curves of the best model (Method 10) presented in Figure 6. Both Methods 9 and 10 employed the AdamW optimizer in PyTorch, with a learning rate of 0.0002 and a weight decay of 0.00001. AdamW is similar to the Adam optimizer, but it decouples weight decay from the learning rate, allowing each parameter to be tuned independently. Cross-entropy loss was used as the loss function, and a step scheduler was employed to decay the learning rate by a factor of 0.1 every five epochs, enabling the model to learn more slowly and deeply. Additionally, average pooling and a dropout of 0.2 were applied. Different from Method 9, which used data augmentation to balance the dataset, Method 10 considered the weight of each class over the total number of images. Figure 6 shows the learning curves for Method 10, using the CL fuzzy texture dataset and pretrained weights from Method 6. The final testing accuracy of this model was 76%. While the training loss curve shows a smooth trend, the other curves exhibited varying degrees of fluctuation across several folds.

Model training was conducted using 5-fold cross-validation and required approximately 45 min on an NVIDIA A100 40GB GPU for the ultrasound dataset (182 images). Each fold used about 145 training images (batch size = 16), yielding around 10 batches per epoch and 20 epochs (around 200 batches per fold), or around 1,000 batches across all folds.

Compared to the CU and CM regions, the classification accuracy for the CL region was generally higher. This disparity may be attributed to the more pronounced degeneration observed in the CL region, which results from increased mechanical and biomechanical stress due to its central role in neck mobility and head support [28,29]. Furthermore, the imbalanced dataset, characterized by a higher proportion of severe cases in the CL region, may have enhanced the classifier’s ability to identify distinctive features, thereby improving its performance.

## 4. Discussion

There are no state-of-the-art algorithms specifically designed for neck disorder assessment systems, making direct comparison difficult. Most previous ultrasound images assessment systems have focused on cancer, specifically tumor detection, whereas this study concentrates on texture-like features related to neck fascia. By applying machine learning techniques to ultrasound images for the assessment of these features, this study explores an area that has not been extensively addressed in the literature.

Azmoodeh-Kalati et al. [30] combined EfficientNetV1 and EfficientNetV2 in an ensemble for breast cancer classification, using ultrasound images. Similarly, Liu et al. [31] introduced pretrained EfficientNetV2 for breast cancer classification. They combined conventional CNNs, such as ResNet_v2 and Inception_v3 with EfficientNetV2 and found that EfficientNetV2-b1 showed the best performance, indicating its effectiveness in the task. Although different breast cancer ultrasound images were used, the outcomes were similar, proving the effectiveness of EfficientNet in medical imaging classification. Given the previous success of applying transfer learning to small datasets, various combinations of transfer learning methods were tested to determine the best model, as shown in Table 2. Due to the small and unbalanced nature of the dataset, it was challenging for EfficientNet to learn potential features directly. Therefore, EfficientNet was first trained using similar, larger datasets, and the learned weights were then transferred to train on the cervical dataset.

**Figure 7:**
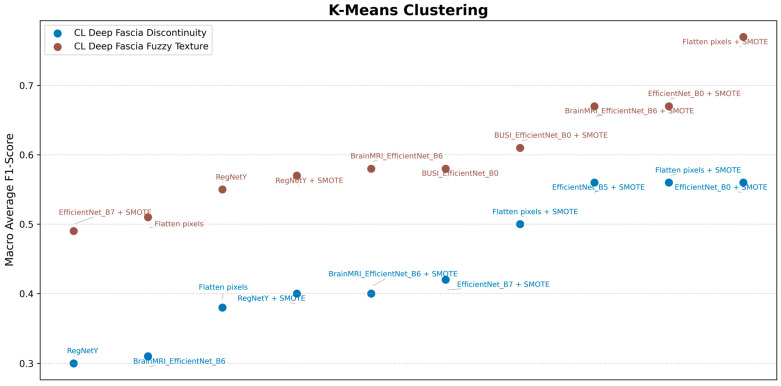
Results of the Conducted Tests. EfficientNet_B7 + SMOTE: Extract features using EfficientNet B7 and then apply SMOTE; EfficientNet_B0 + SMOTE: Extract features using EfficientNet B0 and then apply SMOTE; BUSI_EfficientNet_B0: Extract features using EfficientNet B0 with transfer learnt weights from an online breast ultrasound image dataset; BUSI_EfficientNet_B0 + SMOTE: Extract features using EfficientNet B0 with transfer learnt weights from an online breast ultrasound image dataset and then apply SMOTE; RegNetY: Extract features using RegNetY; RegNetY + SMOTE: Extract features using RegNetY and then apply SMOTE; BrainMRI_EfficientNet_B6: Extract features using EfficientNet B6 with transfer learnt weights from an online brain MRI image dataset; BrainMRI_EfficientNet_B6 + SMOTE: Extract features using EfficientNet B6 with transfer learnt weights from an online brain MRI image dataset and then apply SMOTE.

In addition, preprocessing steps such as Gaussian and median filtering were implemented to normalize texture variance and reduce speckle noise before inputting the data into EfficientNet. To address the computational cost and time constraints, the K-means clustering algorithm was employed to facilitate faster testing with different methods as feature extractors. The tests involved removing the last layer of the neural network to serve as the feature descriptor. The results are shown in Figure 7. K-means clustering was applied to the extracted embedding vectors to partition diagnostic categories efficiently, minimizing redundant computations during inference. This approach was chosen over PCA or direct CNN embeddings because K-means allows grouping of similar feature embeddings into representative cluster centers, enabling faster class decision boundaries without re-training the convolutional layers. Unlike PCA, which performs linear dimensionality reduction, K-means preserves the natural clustering structure of the embeddings, which is critical for maintaining diagnostic category distinctions.

RegNetY, a popular deep learning model, was also tested and compared but did not outperform EfficientNet (as shown in Figure 7). The breast cancer dataset was trained using EfficientNet B0, as the image size was 224 × 224 pixels, and EfficientNet is sensitive to image size. Consequently, other datasets with larger sizes were not used to train EfficientNet B0. For the brain MRI images, which are sized at 512 × 512 pixels, EfficientNet B6 (with images resized to 528 × 528 pixels) and EfficientNet V2L (with images resized to 480 × 480 pixels) were employed, as deeper neural networks tend to learn useful features more effectively.

When compared with EfficientNet B6, EfficientNet V2 demonstrated a 14% improvement in performance on the validation set [17]. The final model, EfficientNet V2L, achieved 96% validation accuracy and 77% testing accuracy. Due to the significant size difference, the breast cancer dataset was not used to train EfficientNet V2L.

Methods 7 and 8 in Table 5 demonstrate the influence of image size on the classification model. In Method 7, the neck ultrasound images were resized to 480 × 480 pixels, while they were resized to 224 × 224 pixels in Method 8. Hence, Methods 7 and 8 yielded insignificant results. Subsequently, development focused on tuning EfficientNet V2L with different parameters. The models were first tuned with various optimizers, including Adam, RMSProp, SGD, and AdamW. Given the small dataset size, overfitting was a concern. The best optimizer, AdamW, was used with a small learning rate of 0.0002. Weight decay (L2 regularization) was applied to prevent overfitting and to encourage the model to focus on more important features. The step scheduler served as a regularization and fine-tuning technique to help the model converge for better feature learning. Additionally, average pooling with a 20% dropout was applied to regularize and prevent overfitting. All combinations of the different regularization techniques were tested, and the best combination was reported in the result. After testing the best combinations of the optimizers, Method 9 did not yield satisfactory results. Due to the highly unbalanced dataset, the augmented images created were in a big amount compared to the normal cases, creating a bias in the model. To further minimize this bias, the weighted random sampler from PyTorch was used in Method 10, also referred to as the final model. This method used data based on the weight of the particular class, thereby ensuring that each class was represented proportionally during training. This approach reduces the impact of the higher selection probability for the majority class.

**Figure 8:**
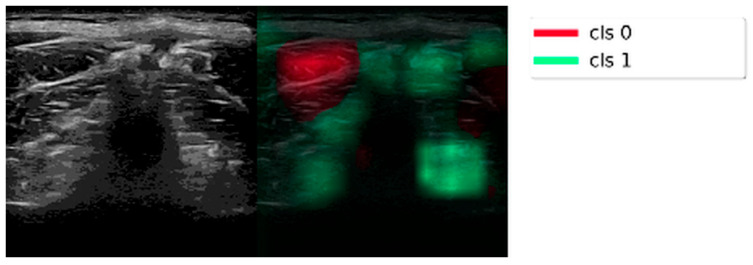
The Sample Output of Grad-CAM. It shows how the model determines which parts of the input are more relevant to the features of class 0 or class 1. In this sample, only the red highlighted part led the model to classify it as class 0. (Note: All four features—Deep Fascia Discontinuity, Deep Fascia Fuzzy Texture, Deep Fascia and Myofascial Adhesion, MHS—are evaluated based on the same ultrasound image, so only one Grad-CAM visualization is shown).

The inference of the proposed algorithm was visualized using Gradient-weighted Class Activation Mapping (Grad-CAM), a technique used to visualize the regions of an image important for predicting a particular class label by examining the last feature layer [32]. A sample output is shown in Figure 8, demonstrating that the model did not learn the noisy parts, unlike in K-means clustering. Recent research in medical image analysis has demonstrated the benefit of hybrid approaches that combine deep learning with handcrafted feature representations. For example, hybrid models integrating CNNs with handcrafted texture features have shown enhanced performance in histopathological diagnosis tasks, such as malignant lymphoma classification [33], and in pneumonia detection from chest radiographs, where texture descriptors like GLCM complement CNN-based features to improve robustness [34]. These findings conceptually support our use of texture-oriented characterization alongside deep feature learning, adapted here to address the challenge of cervical fascia analysis.

The observed performance variation across cervical regions likely reflects inherent anatomical and biomechanical differences rather than limitations of the modeling approach [35]. The lower cervical region plays a primary role in load transmission and mechanical stabilization of the neck and is characterized by relatively well-defined and continuous fascial structures, which may yield more consistent texture patterns for feature learning. In contrast, the middle and upper cervical regions comprise anatomically intricate soft-tissue arrangements with overlapping fascia, musculature, and connective tissues that support fine motor control and multidirectional movement. Such structural complexity can result in more heterogeneous texture representations, making discrimination between diagnostic categories inherently more challenging. Similar region-dependent performance variations have been reported in recent medical imaging studies, where deep learning models demonstrated differing classification and segmentation accuracy across anatomically distinct subregions. These findings suggest that region-specific anatomical characteristics are an important consideration when interpreting model performance in ultrasound-based musculoskeletal analysis.

## 5. Limitations and Recommendations

The neck disorder assessment system is new in the medical intelligence field, unlike cancer assessment systems, which have been extensively researched. The main challenge in developing this system lies in the limitation of the dataset, which is small, highly imbalanced, and lacks available open datasets for transfer learning. While many state-of-the-art algorithms perform well on small datasets, they typically require large datasets for effective transfer learning. The ideal public dataset should be large in size, with consistent pixel size and texture-related images. Due to the limited public dataset options, the breast cancer and brain MRI datasets used in this study were the best options available, despite being tumor-related and having smaller pixel sizes.

Additionally, since the dataset was collected and analyzed by only two clinicians from a single center, it limits the generalizability and robustness of the algorithm. Consequently, the solution may not be objective enough to assist clinicians without bias. This limitation underscores the importance of multi-center data integration in medical imaging–based artificial intelligence research. For instance, Ghabri et al. [36] applied ImageNet-pretrained CNNs with transfer learning to classify fetal ultrasound images, using datasets collected from multiple hospitals across countries such as Spain, Egypt, and Algeria. They demonstrated the importance of using robust datasets to produce a robust classification model, ensuring the solution is suitable for a broader population. Accordingly, a key future direction of this work is to expand data collection to include multi-center and multi-operator datasets, enabling the model to better capture inter-institutional and inter-population variability. Furthermore, prospective multi-center validation studies would be essential to evaluate the stability of model performance over time, support clinical translation, and ensure that the proposed framework remains reliable and effective across diverse real-world clinical settings.

Besides that, with the capability of Generative AI, these datasets can be combined to create a larger dataset for synthetic image generation using generative deep learning models. Specifically, diffusion-based methods (e.g., denoising diffusion models shown to improve diversity in cardiac ultrasound) or GAN-based techniques (such as CycleGAN architectures tailored for multi-organ ultrasound enhancement) could be employed to ensure reproducibility and high-quality image synthesis [37,38]. If successful, synthetic image generation could contribute to open datasets like MedMNIST, accelerating the development of texture-related ultrasound image assessment systems. While Grad-CAM offered an initial view of how the model interprets ultrasound features, its explanatory capability remains limited. In future work, more robust interpretability methods such as Shapley Additive Explanations (SHAP) and Local Interpretable Model-Agnostic Explanations (LIME), which can provide clearer, model-agnostic insights into the features driving the model’s decisions [39], should be employed. Grad-CAM++ will also be considered to improve localization and highlight finer anatomical details. These enhancements aim to address current limitations and support greater transparency and confidence when applying the model in clinical practice.

Moreover, future studies should aim to obtain segmented masks from clinicians as the ground truth. Each cervical part has several categories, each focusing on different regions in the ultrasound image. Ground truth labels of segmented masks or regions will aid in feature extraction and classification algorithms. The absence of such region-specific annotations in the current study restricts the model’s ability to explicitly attend to anatomically relevant fascia structures and may partly explain the moderate performance observed in certain cervical regions and feature categories. Excellent zero-shot segmentation models such as Medical Segment Anything Model (MedSAM) show promising performance. The mask region will help the algorithm focus on specific regions for each category, enhancing the significance of the features. Future work could also explore alternative deep learning architectures, including lightweight CNNs and CNN-ViT models, along with a systematic comparison of computational efficiency, to further enhance both diagnostic accuracy and efficiency in medical imaging.

To make the model more clinically meaningful and adaptable to real-world cases, future work should consider incorporating additional patient information such as age, BMI, palpation findings, and relevant medical history. Bringing these elements together through a multimodal fusion approach would allow the model to capture a fuller picture of each patient, ultimately improving its robustness and ability to handle natural variations between individuals. In addition, future work can include a full ablation study to quantitatively evaluate the contribution of each component of the model.

Finally, while developing and sharing ultrasound datasets is critical for advancing medical AI, ethical and privacy considerations must be prioritized. Even de-identified images carry potential re-identification risks if metadata is not carefully managed. All data sharing should follow robust governance, ensuring patient confidentiality, informed consent, and responsible stewardship. Transparent consent processes and secure data handling are essential to respect patient rights while maximizing the societal benefit of data reuse [40,41].

## 6. Conclusions

This study demonstrated the potential of EfficientNet-based deep learning models for automated neck disorder assessment using ultrasound images. The final model, developed with transfer learning and validated through 5-fold cross-validation and an independent clean test dataset, showed its strongest performance in the lower cervical region. Specifically, deep fascia fuzzy texture and myofascial adhesion achieved weighted F1-scores of 76% and 81%, indicating significant diagnostic value for clinical support. These results highlight the model’s potential for clinical use in scenarios such as screening and triage, where it could enable rapid, consistent assessments, leading to early identification and prioritization of neck disorders. However, the study is limited by a small, imbalanced dataset and the lack of segmentation masks, which may impact generalizability and feature localization. Future work should focus on expanding the dataset across multiple centers to enhance generalizability and incorporating segmentation masks for region-specific learning. Additionally, advanced augmentation or generative techniques could be explored to further improve model robustness. Overall, this model lays a promising foundation for supporting neck disorder assessment, offering the potential to improve the consistency, efficiency, and accuracy of clinical ultrasound interpretation.

## Figures and Tables

**Table 1:** Summary of Patients’ Information.

Variable	Gender		
	Male (*n* = 75)	Female (*n* = 109)	*p*-Value
Age, years	42.81 ± 15.60	40.35 ± 14.15	0.267
Weight, kg	74.19 ± 14.14	59.31 ± 12.85	<0.001
Height, cm	170.45 ± 7.12	159.75 ± 5.87	<0.001
BMI, kg/m^2^	26.26 ± 4.67	23.64 ± 4.87	<0.001

Note: The scores are in the form of average ± standard deviation.

**Table 2:** Number of Images for Each Score in Each Category.

Features	Class	Cervical Lower (CL)	Cervical Middle (CM)	Cervical Upper (CU)
Deep fascia discontinuity	0	14	23	23
	1	34	85	71
	2	134	74	88
Deep fascia fuzzy texture	0	37	70	79
	1	145	112	103
Modified Heckmatt scale (MHS)	1	37	52	55
	2	74	84	78
	3	60	36	42
	4	11	10	7
Deep fascia and myofascial adhesion	0	28	95	94
	1	154	87	88

**Table 3:** Methods Used for Classification Models.

Method	Model	Dataset	Weight Used
1	EfficientNet B0	Breast Cancer	ImageNet (1000 classes)
2	ResNet 101	Brain MRI (reduced size)	ImageNet (1000 classes)
3	EfficientNet B6	Brain MRI (reduced size)	ImageNet (1000 classes)
4	EfficientNet V2L	Brain MRI (reduced size)	ImageNet (1000 classes)
5	EfficientNet V2L	Brain MRI (full size)	ImageNet (1000 classes)
6	EfficientNet V2L (with dropout)	Brain MRI (full size)	ImageNet (1000 classes)
7	EfficientNet V2L	Neck Disorder (Augmented)	ImageNet (1000 classes)
8	EfficientNet B0	Neck Disorder (Augmented)	Breast Cancer + EfficientNet B0
9	EfficientNet V2L	Neck Disorder (Augmented)	Brain MRI (full size) + EfficientNet V2L
10	EfficientNet V2L	Neck Disorder (weighted)	Brain MRI (full size) + EfficientNet V2L

**Table 4:** Training Results of Different Classification Models on Open Datasets.

Method	Number of Parameters (Millions)	Breast Cancer Dataset	Brain MRI (Reduced)	Brain MRI (Full)
1	5.3	0.99	NA *	NA *
2	44.5	NA *	0.85	
3	43.0		0.53	
4	118.5		0.67	
5	118.5		NA *	0.72
6	118.5			0.96

Note: NA *: Not Applicable (configurations that were not evaluated due to incompatible input image resolutions for the corresponding model architecture). Method 1: EfficientNet B0 with breast cancer dataset and pretrained weight with 1000 classes ImageNet; Method 2: ResNet 101 with reduced size brain MRI dataset and pretrained weight with 1000 classes ImageNet; Method 3: EfficientNet B6 with reduced size brain MRI dataset and pretrained weight with 1000 classes ImageNet; Method 4: EfficientNet V2L with reduced size brain MRI dataset and pretrained weight with 1000 classes ImageNet; Method 5: EfficientNet V2L with full size brain MRI dataset and pretrained weight with 1000 classes ImageNet; Method 6: EfficientNet V2L with dropout and full size brain MRI dataset and pretrained weight with 1000 classes ImageNet.

**Table 5:** The Overall Performance of The Classification Algorithm.

Method	F1-Score	Deep Fascia Discontinuity (%)			Deep Fascia Fuzzy Texture (%)			Deep Fascia and Myofascial Adhesion (%)			MHS (%)
		CL	CM	CU	CL	CM	CU	CL	CM	CU	
7	Macro average	45	51	49	53	52	55	NS			NS
	Weighted average	52	47	49	55	52	55				
8	Macro average	NS			51	50	52	NS			
	Weighted average				53	50	52				
9	Macro average	NS			62	61	64	NS			
	Weighted average				62	61	64				
10	Macro average	35	67	65	74	72	49	78	55	54	
	Weighted average	52	63	65	76	72	49	81	55	54	

Note: NS: Not Significant (non-convergent training); CL: Cervical Lower region; CM: Cervical Middle region; CU: Cervical Upper region; Method 7: EfficientNet V2L directly trained on the given dataset with augmentation with the original pretrained weight of 1000 classes ImageNet; Method 8: EfficientNet B0 trained on the given dataset with augmentation with pretrained weight using the breast cancer dataset which was trained on EfficientNet B0 with weight of 1000 classes ImageNet; Method 9: EfficientNet V2L trained on the given dataset with augmentation to balance the dataset, with pretrained weight using full-size brain MRI dataset which was trained on EfficientNet V2L with weight of 1000 classes ImageNet; Method 10: EfficientNet V2L trained on the given dataset with weighted random sampler, with pretrained weight using full-size brain MRI dataset which was trained on EfficientNet V2L with weight of 1000 classes ImageNet.

**Figure A1:**
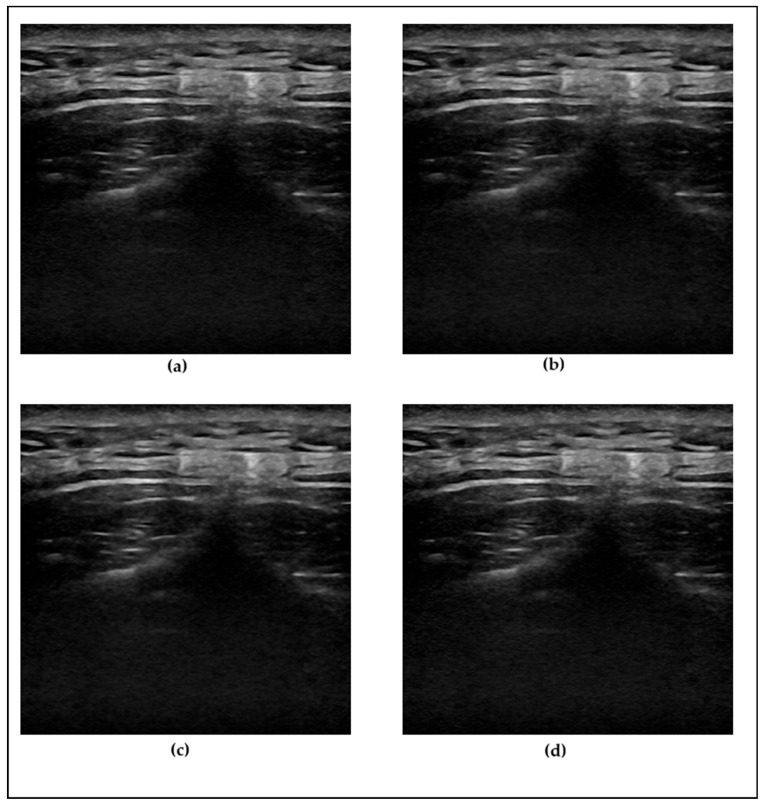
Comparison of image filtering techniques. (**a**) Raw image without filtering; (**b**) Median filter applied, yielding a PSNR of 43.14; (**c**) Bilateral filter applied, yielding a PSNR of 42.04; (**d**) Gaussian filter applied, achieving the highest PSNR of 49.16.

**Figure A2:**
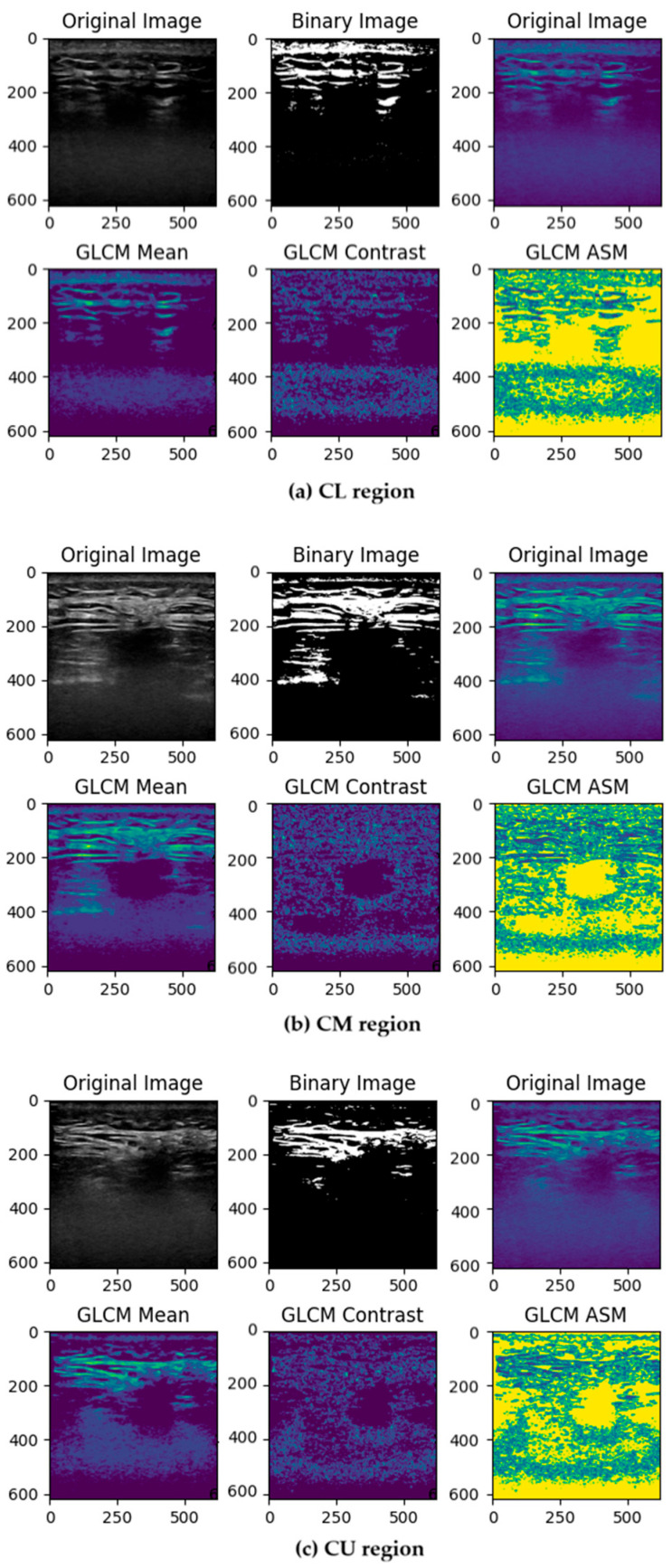
Additional representative ultrasound samples demonstrating GLCM texture characterization, i.e., (**a**) Cervical lower (CL) region, (**b**) Cervical middle (CM) region, (**c**) Cervical upper (CU) region. Quantitative GLCM metrics show consistent reductions in entropy and increases in variance across the displayed images, supporting the effectiveness of the filtering process in suppressing noise while maintaining muscle fiber texture integrity.

## Data Availability

The study’s data and resources are presently being analyzed and cannot yet be accessed by the public. We are unable to offer more details or access to the data currently due to the ongoing nature of the research.

## Abbreviations

The following abbreviations are used in this manuscript:

ACGAN	Auxiliary Classifier Generative Adversarial Network
BMI	Body Mass Index
CL	Cervical Lower region
CM	Cervical Middle region
CROM	Cervical Range of Motion
CT	Computed Tomography
CU	Cervical Upper region
FN	False Negative
FP	False Positive
GLCM	Gray-Level Co-Occurrence Matrix
Grad-CAM	Gradient-weighted Class Activation Mapping
MHS	Modified Heckmatt Scale
MRI	Magnetic Resonance Imaging
OBNLM	Optimized Blockwise Non-local Means
PSNR	Peak Signal-to-Noise Ratio
TN	True Negative
TP	True Positive
